# Supplementary material for: An Endoglucanase Secreted by Ustilago esculenta Promotes Fungal Proliferation
Source: J Fungi (Basel). 2022 Oct 7;8(10):1050. doi: 10.3390/jof8101050 (PMC9605326; doi:10.3390/jof8101050)
Supplement: Supplementary file 1 [file jof-08-01050-s001.zip › suplementary Materials.pdf]

An Endoglucanase Secreted by *Ustilago esculenta* Promotes Fungal Proliferation

Zhongjin Zhang, Jiahui Bian, Yafen Zhang *, Wenqiang Xia, Shiyu Li and Zihong Ye *

Supplementary information

Figure S1: *UeEgl1* deletion verification;

Figure S2: *UeEgl1* expressed differentially in T-type and MT-type of *U. esculenta*;

Figure S3: The expression patterns of *UeEgl1* at budding, filamentous growth, and pathogenic development stages;

Figure S4: Transcriptome analysis of *Z. latifolia* infected with WT, KO, or OE at 0 and 3 dpi;

Figure S5: Differentially expressed genes (DEGs) of *Z. latifolia* were enriched and classified by the Kyoto encyclopedia of genes and genomes (KEGG) pathways;

Table S1: Primers used in this study.

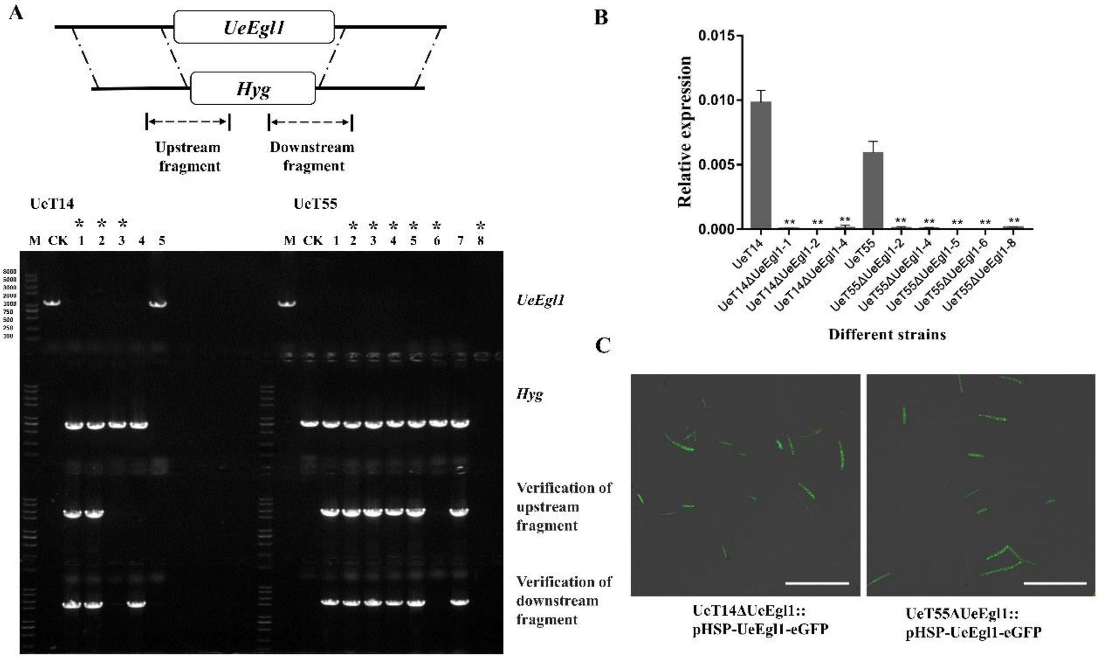

Figure S1. Verification of *UeEgl1* deletion and over-expressed strains. (**A**) Specific fragment verification. M: Trans2k plus Ⅱ DNA marker; CK: *UeEgl1* fragment positive control. UeT14 lane 1–5: UeT14ΔUeEgl1 deletion strains; UeT55 lane 1–8: UeT55ΔUeEgl1 deletion strains. "*" represent candidates of *UeEgl1* mutants. (**B**) qRT-PCR verification of *UeEgl1* deletion strains. The relative expression level of *UeEgl1* was used *β-actin* as an internal reference gene. "**" indicate a significant reduction of *UeEgl1* expression in *UeEgl1* deletion mutants (Student's t-test, $p < 0.01$). (**C**) UeEgl1 localization and *UeEgl1* over-expressed verification. Three transformants for each strain of eGFP tagged UeEgl1 under the control of endogenous strong promoter *HSP70* were observed by confocal microscopy. Bar = 25 μm.

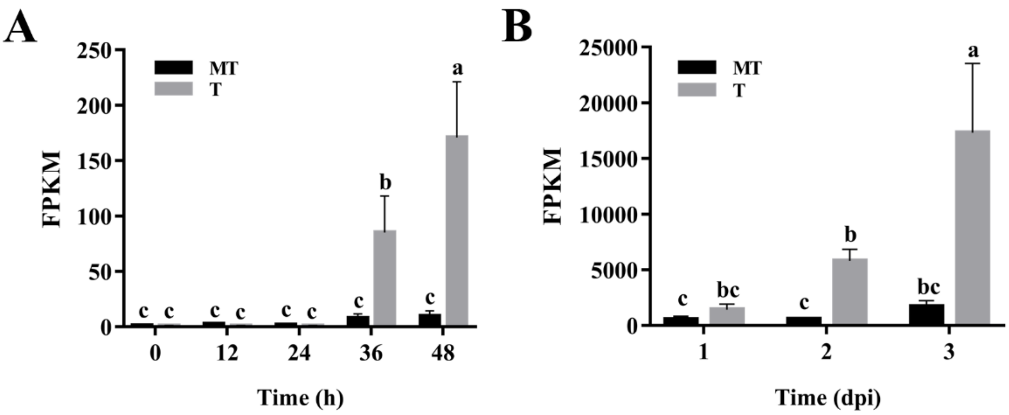

Figure S2. *UeEgl1* expressed differentially in T-type and MT-type of *U. esculenta*. (**A**) *UeEgl1* fragments per kilobase of exon model per million mapped fragments (FPKM) of MT-Type and T-type at 0, 12, 24, 36, 48 h of in vitro mating experiment. (**B**) *UeEgl1* FPKM of T-type and MT-type at 1, 2, 3 dpi. Different letters above the columns represent significant differences at the analysis of variance (ANOVA) ($p < 0.05$).

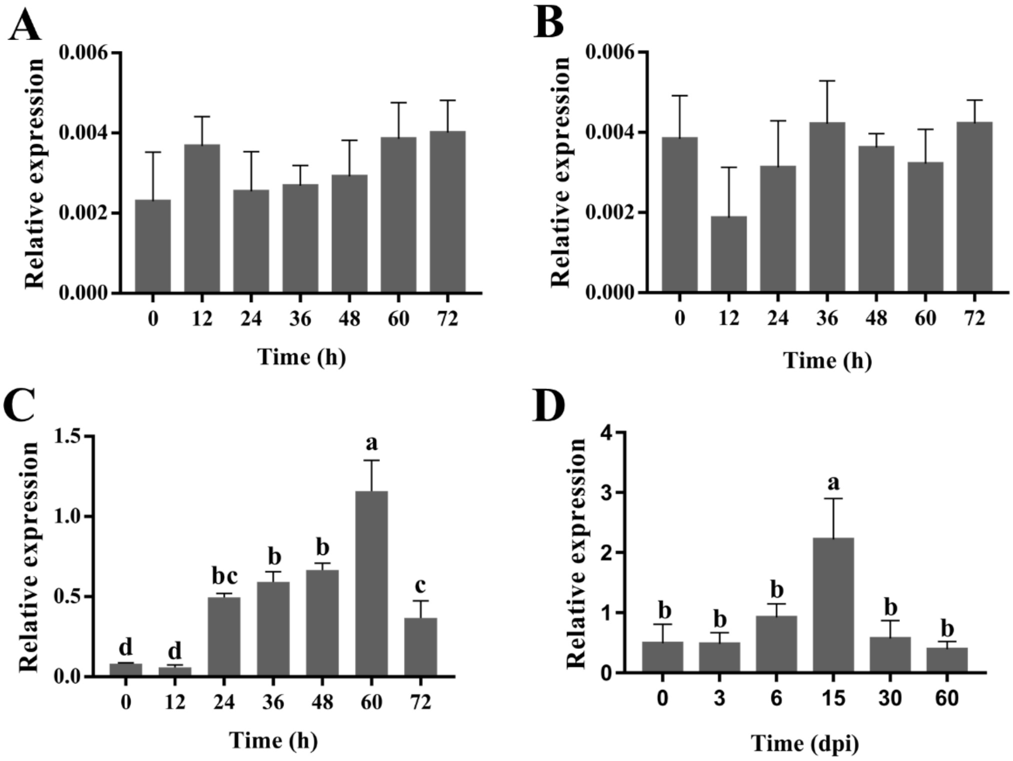

Figure S3. The expression patterns of *UeEgl1* at budding, filamentous growth, and pathogenic development stages. (**A**,**B**) *UeEgl1* relative expression of haploid UeT10 and UeT46 strains every 12 h on YESP medium, respectively. (**C**) *UeEgl1* of MT-type relative expression every 12 h after mating on YESP medium. Different letters above the columns represent significant differences at the analysis of variance (ANOVA) ($p < 0.05$). (**D**) Plant samples infected with merged UeT10 and UeT46 were collected at 0, 3, 6, 15, 30, and 60 days, then *UeEgl1* relative expression was analyzed. Different letters above the columns represent significant differences at the analysis of variance (ANOVA) ($p < 0.05$). The expression of *UeEgl1* was related to the expression of *β-actin*,

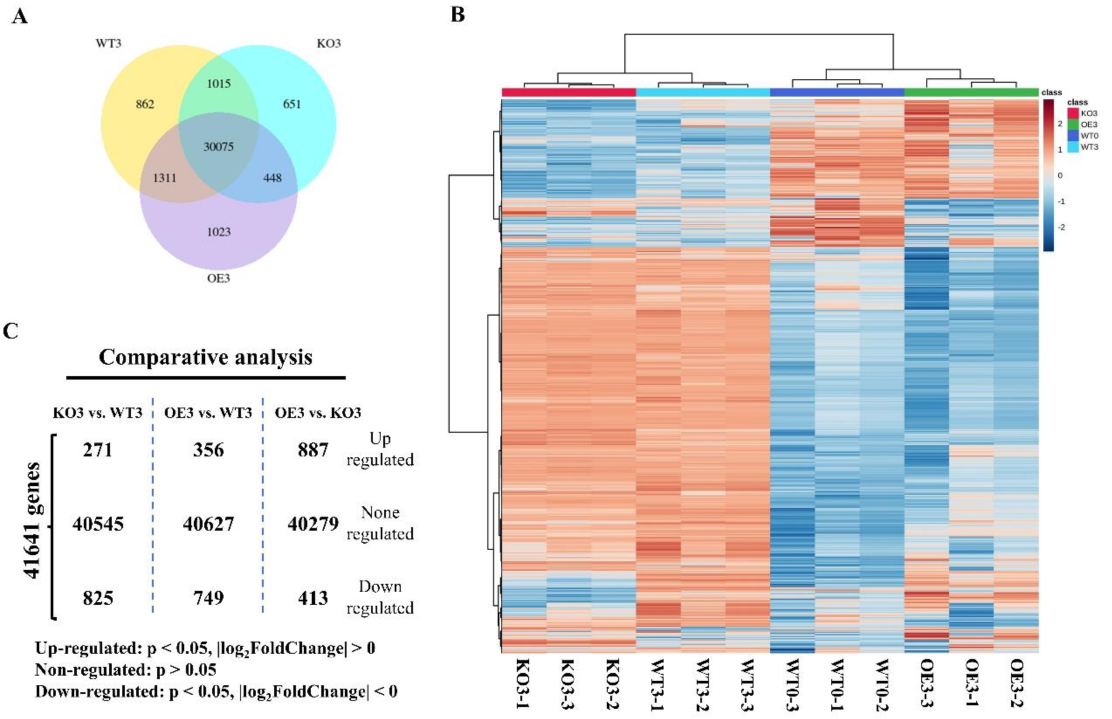

Figure S4. Transcriptome analysis of *Z. latifolia* infected with WT, KO, or OE at 0 and 3 dpi. (**A**) Transcriptome profiles of differentially expressed genes (DEGs) of *Zizania latifolia* infected by WT, or KO, or OE at 3 dpi. (**B**) Differentially expressed genes expression heatmap. The expression level was compared to the general mean by $\log_{10}$(foldchange). Up-regulated genes: light red to deep red; equally regulated genes: white; down-regulated: light blue to deep blue. (**C**) A total of 41,641 differentially expressed genes in WT3, KO3, and OE3 were compared.

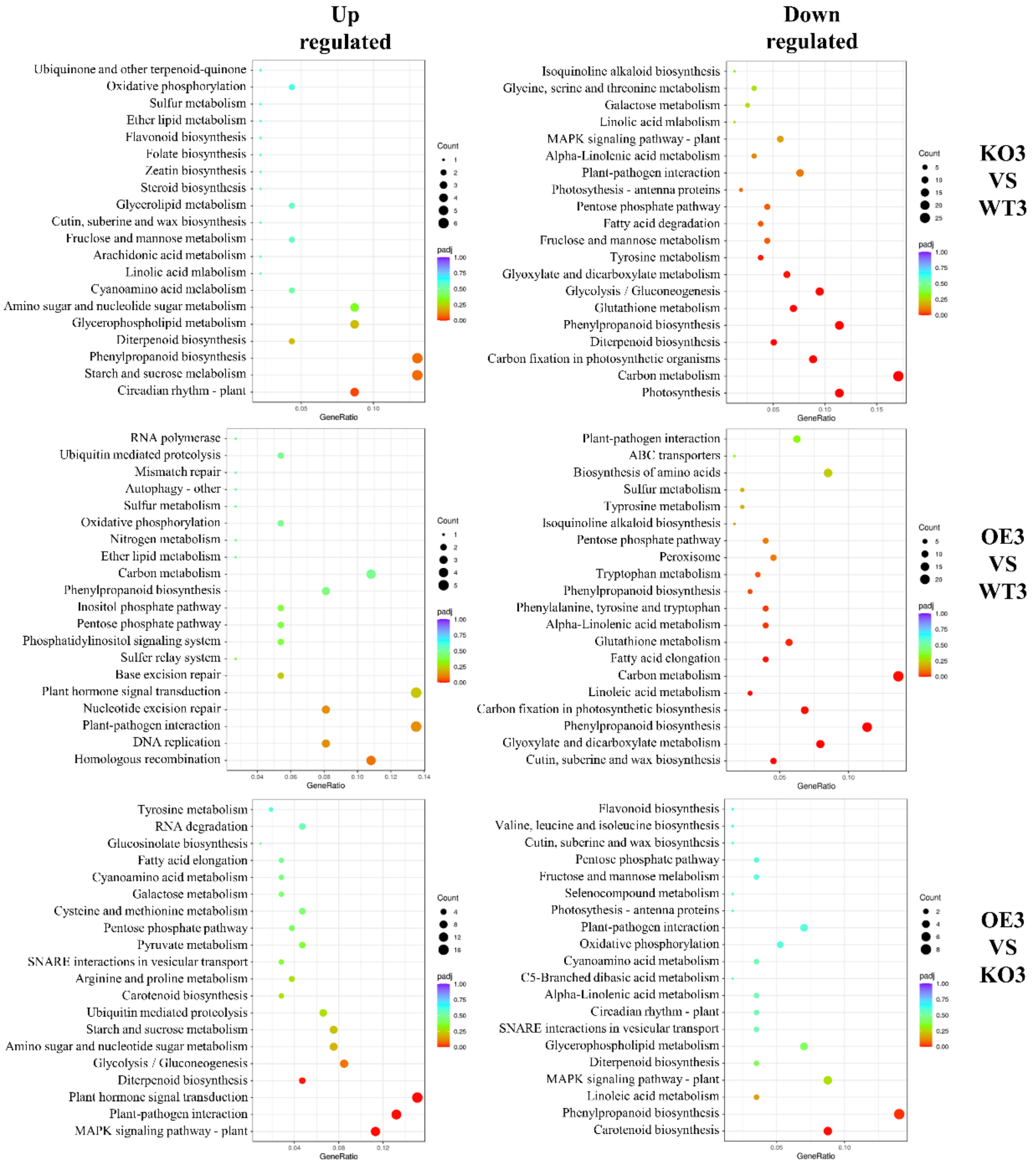

Figure S5. Differentially expressed genes (DEGs) of *Z. latifolia* were enriched and classified by the Kyoto encyclopedia of genes and genomes (KEGG) pathways. KO3 versus WT3, OE3 versus WT3, and OE3 versus KO3 were compared. Images on the left were up-regulated DEGs of KEGG pathways, and images on the right were down-regulated DEGs of KEGG pathways. The size of the dot represents gene number, color represents the corrected *p*-value (padj) range.

Table S1. primers used in this study.

Primer	Sequence (5’-3’)	Use
egl1-gF	CCGCTTGATTCATCGTGTCTG	*UeEgl1* cloning
egl1-gR	TTTCGGGATGGAAACGACTG	
egl1-cF	ATGTCGTTCAAACTCAAGG	
egl1-cR	TCAGTGCTTGTTCTTGCAG	
egl1-F-f	GTGAATTCGAGCTCGGTACCGTCCAGCTGGACCTCTGACT	*UeEgl1* knockout Vector construction
egl1-F-r	TCTAGAGGATCCCCGGTACCGGCGAAGATAAACAGAGAAA	
egl1-R-f	CGTCGACCTGCAGGCATGCAGCTAGACTGCCATTCGCATA	
egl1-R-r	GACCATGATTACGCCAAGCTGATTACGCCAAGCTTGCATG	
Hyg3	GGATGCCTCCGCTCGAAGTA	*UeEgl1* deletion verification
Hyg4	CGTTGCAAGACCTGCCTGAA	
Hyg-YZ-F	TCGTTATGTTTATCGGCACT	
Hyg-YZ-R	TCGGCGAGTACTTCTACACA	
egl1-QF	CTGGCTTTCGGCTTTGCT	Quantitative PCR validation
egl1-QR	CGTTGGTCACCTGGAAGATG	
β-actin-QF	CAATGGTTCGGGAATGTGC	
Zl-actin- qF	GACGGTGAGGATATCAAGCC	
Zl-actin- qR	GCGAGGGCAACCGACAATAC	
HSP-egl1-F	GCCTTAGAATCGTCATCCCCATGTCGTTCAAACTCAACGT	*UeEgl1* overexpression Vector construction
HSP-egl1-R	TCCTCGCCCTTGCTCACCATGTGCTTGTTCTTGCAGAACT	
HSP-YZ-F	GAACTCGAGCAGCTGAAGCT	*UeEgl1* overexpression verification
HSP-YZ-R	CGCTGAACTTGTGGCCGTTT	
