# Supplementary material for: An Endoglucanase Secreted by Ustilago esculenta Promotes Fungal Proliferation
Source: J Fungi (Basel). 2022 Oct 7;8(10):1050. doi: 10.3390/jof8101050 (PMC9605326; doi:10.3390/jof8101050)
Supplement: Supplementary file 1 [file jof-08-01050-s001.zip › Table S1.pdf]

Table S1. primers used in this study.

Primer	Sequence (5'-3')	Use
egl1-gF	CCGCTTGATTCATCGTGTCTG	*UeEgl1* cloning
egl1-gR	TTTCGGGATGGAAACGACTG	
egl1-cF	ATGTCGTTCAAACTCAAGG	
egl1-cR	TCAGTGCTTGTTCTTGCAG	
egl1-F-f	GTGAATTCGAGCTCGGTACCGTCCAGCTGGACCTCTGACT	*UeEgl1* knockout Vector construction
egl1-F-r	TCTAGAGGATCCCCGGTACCGGCGAAGATAAACAGAGAAA	
egl1-R-f	CGTCGACCTGCAGGCATGCAGCTAGACTGCCATTCGCATA	
egl1-R-r	GACCATGATTACGCCAAGCTGATTACGCCAAGCTTGCATG	
Hyg3	GGATGCCTCCGCTCGAAGTA	*UeEgl1* deletion verification
Hyg4	CGTTGCAAGACCTGCCTGAA	
Hyg-YZ-F	TCGTTATGTTTATCGGCACT	
Hyg-YZ-R	TCGGCGAGTACTTCTACACA	
egl1-QF	CTGGCTTTCGGCTTTGCT	Quantitative PCR validation
egl1-QR	CGTTGGTCACCTGGAAGATG	
β-actin-QF	CAATGGTTCGGGAATGTGC	
Zl-actin- qF	GACGGTGAGGATATCAAGCC	
Zl-actin- qR	GCGAGGGCAACCGACAATAC	
HSP-egl1-F	GCCTTAGAATCGTCATCCCCATGTCGTTCAAACTCAACGT	*UeEgl1* overexpression Vector construction
HSP-egl1-R	TCCTCGCCCTTGCTCACCATGTGCTTGTTCTTGCAGAACT	
HSP-YZ-F	GAACTCGAGCAGCTGAAGCT	*UeEgl1* overexpression verification
HSP-YZ-R	CGCTGAACTTGTGGCCGTTT	
