# Supplementary figures and images for: An Endoglucanase Secreted by Ustilago esculenta Promotes Fungal Proliferation
Source: J Fungi (Basel). 2022 Oct 7;8(10):1050. doi: 10.3390/jof8101050 (PMC9605326; doi:10.3390/jof8101050)

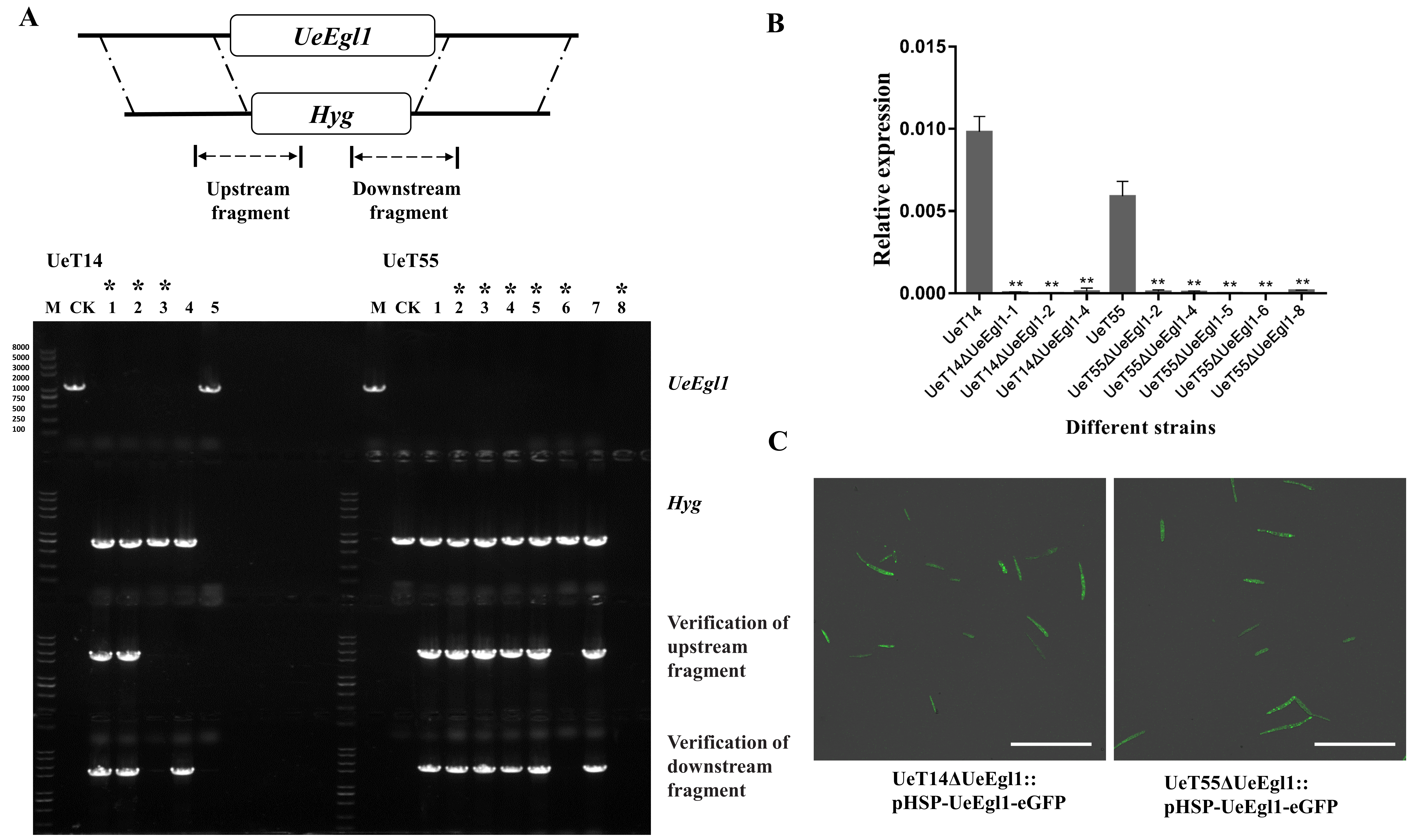

Supplement: Supplementary file 1 [file jof-08-01050-s001.zip › Figure S1.tif]

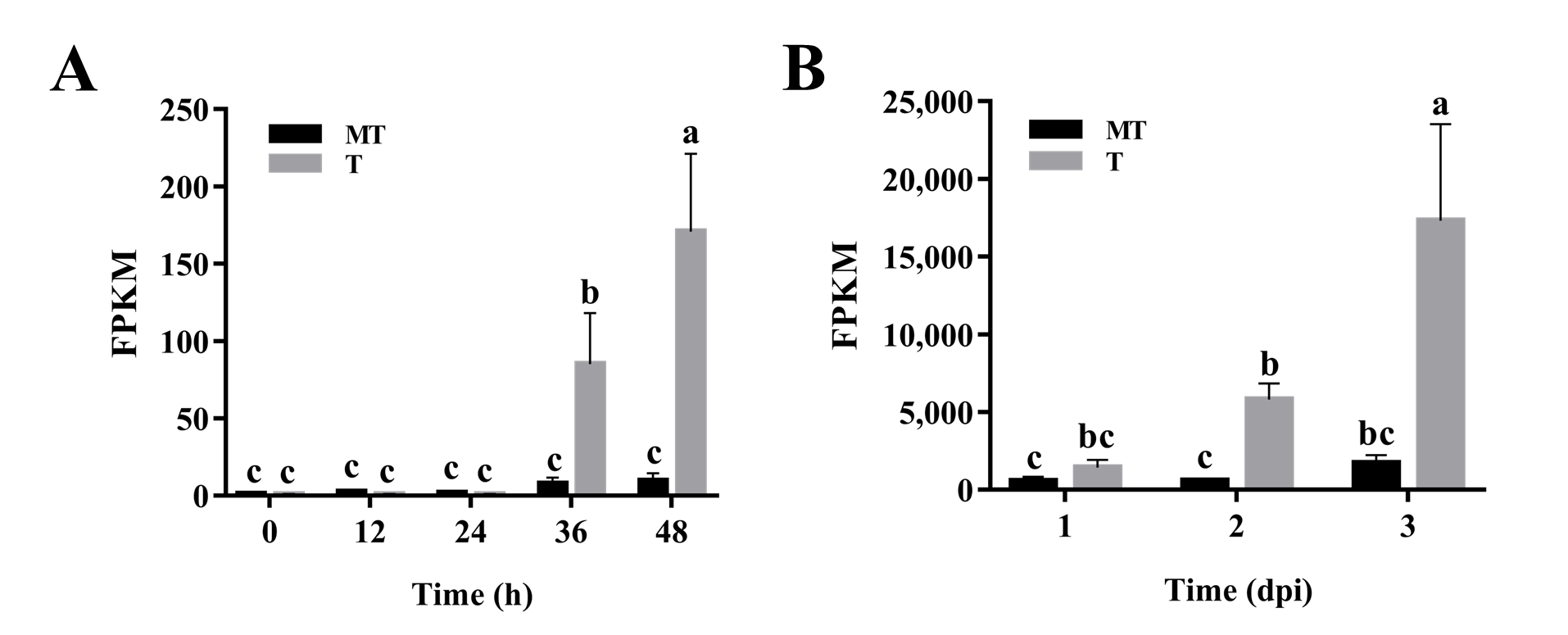

Supplement: Supplementary file 1 [file jof-08-01050-s001.zip › Figure S2.tif]

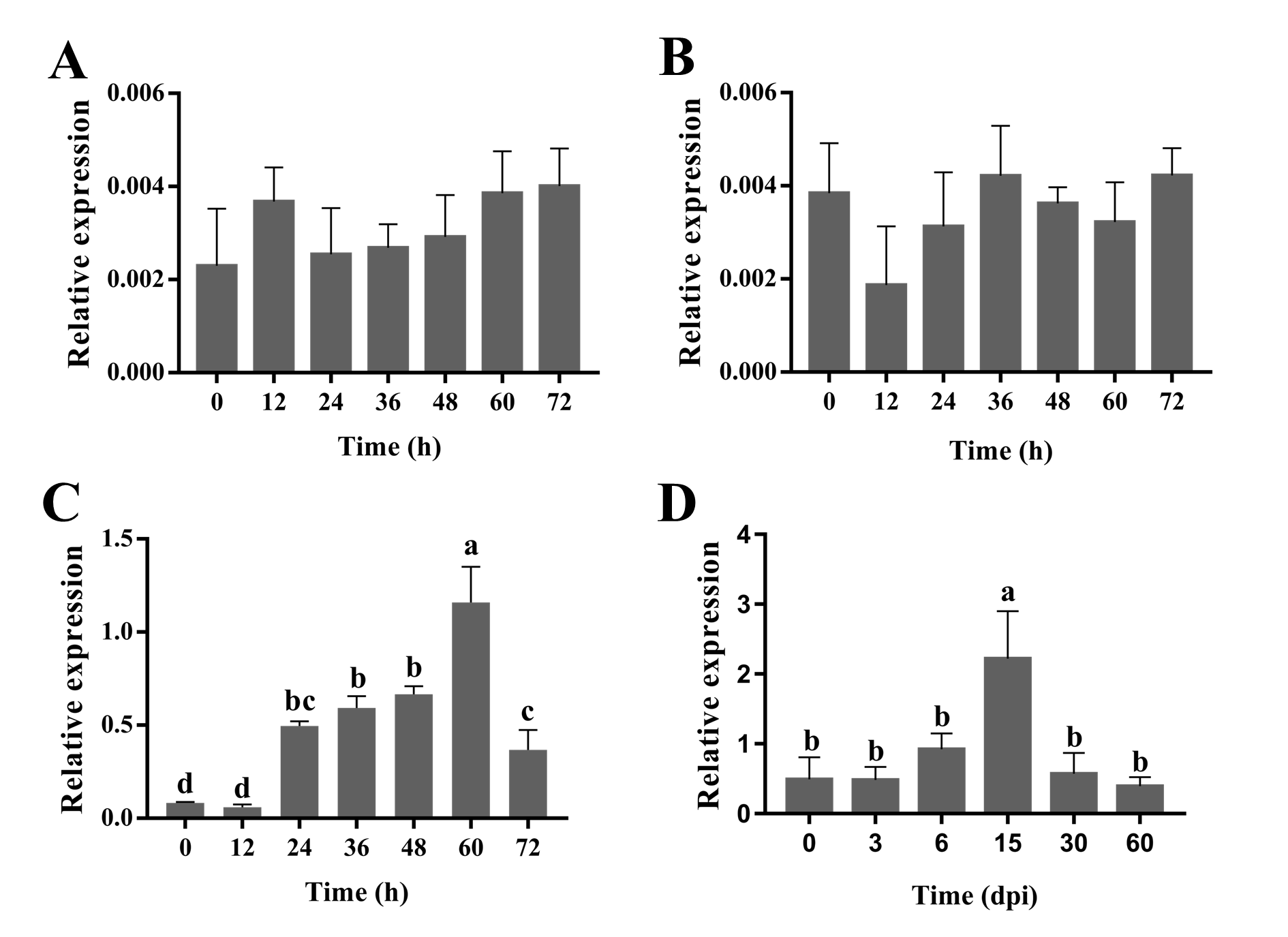

Supplement: Supplementary file 1 [file jof-08-01050-s001.zip › Figure S3.tif]

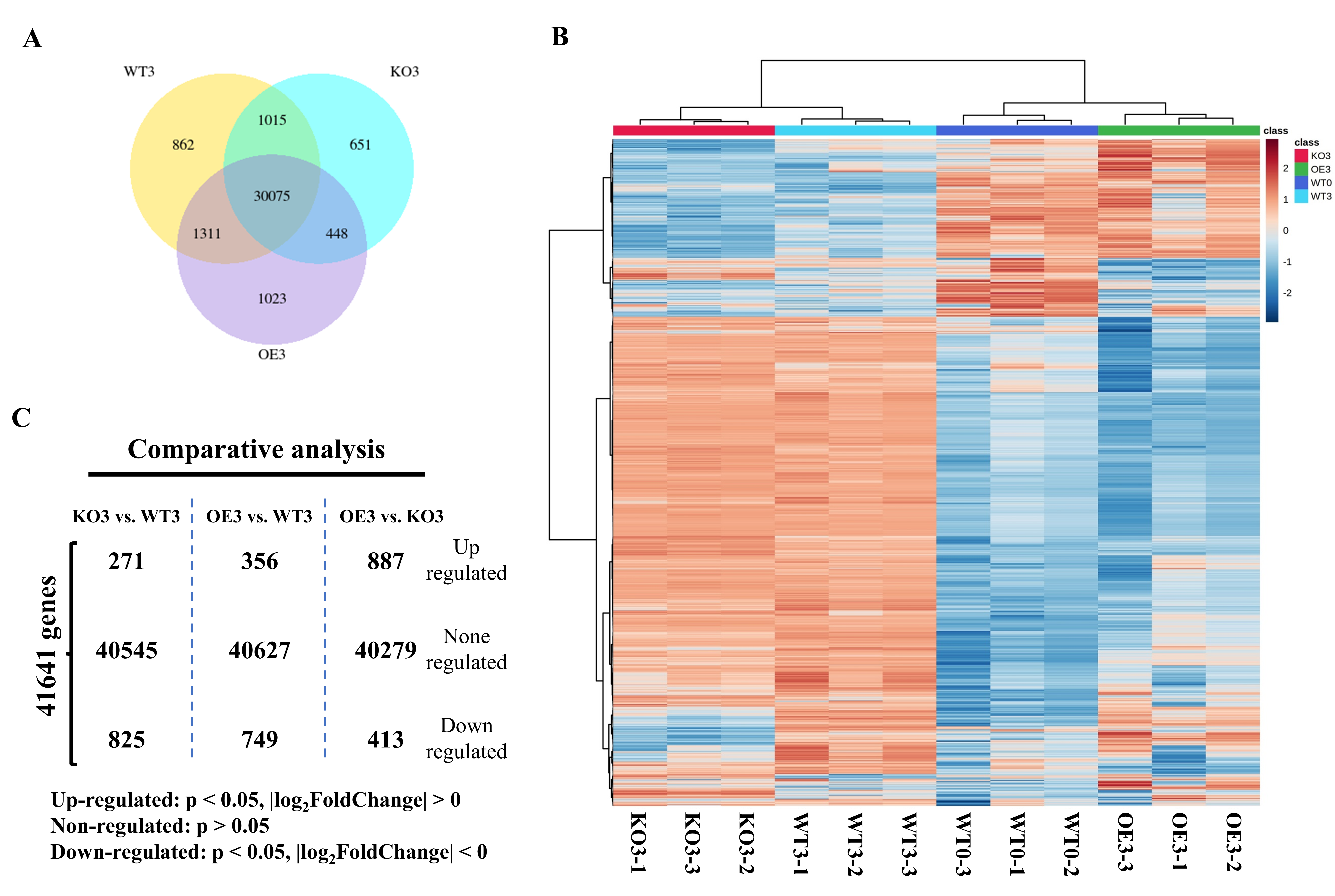

Supplement: Supplementary file 1 [file jof-08-01050-s001.zip › Figure S4.tif]

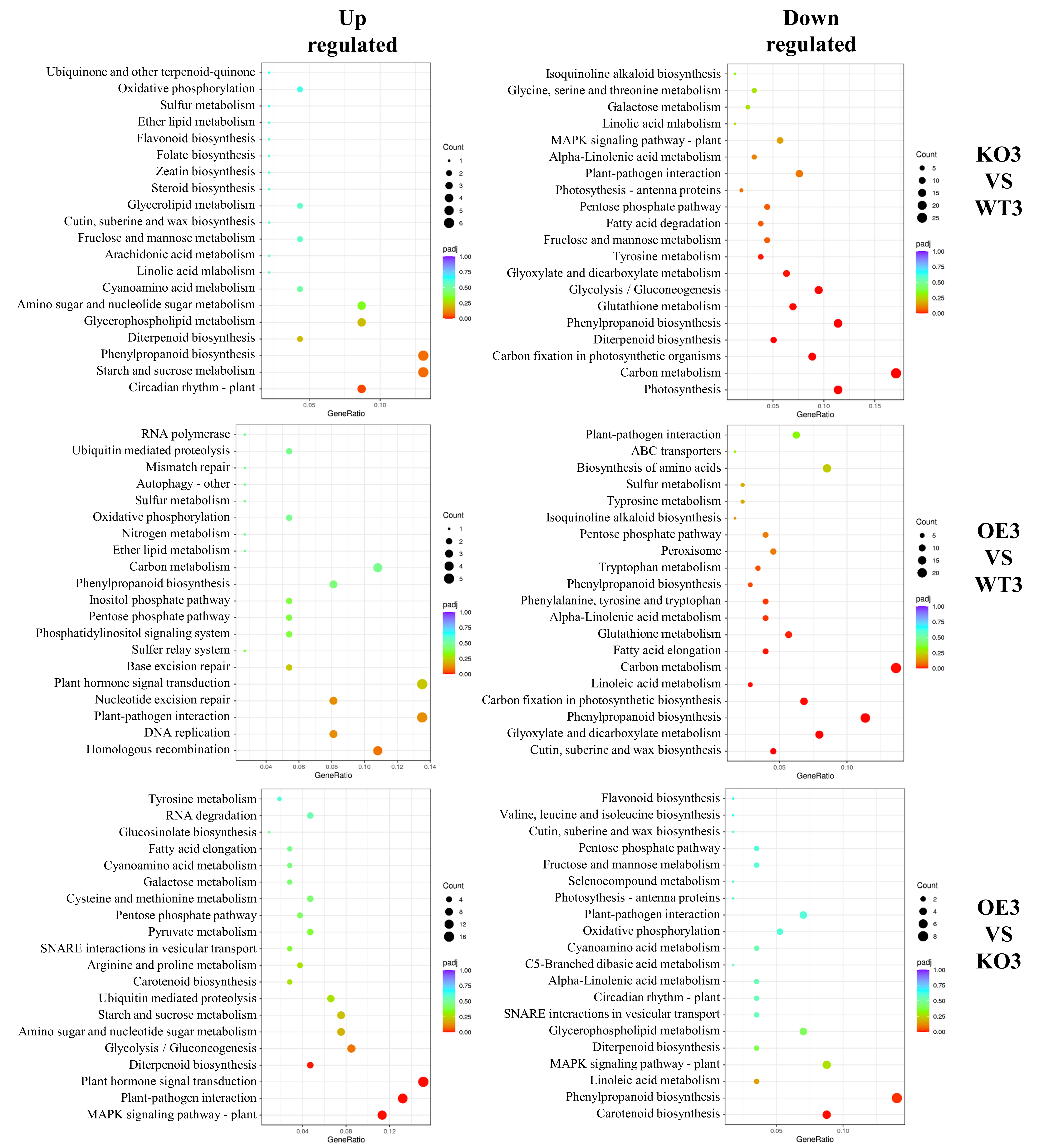

Supplement: Supplementary file 1 [file jof-08-01050-s001.zip › Figure S5.tif]
